# Transfection of *Culicoides sonorensis* biting midge cell lines with *Wolbachia pipientis*

**DOI:** 10.1186/s13071-019-3716-0

**Published:** 2019-10-15

**Authors:** Arnab Ghosh, Dane Jasperson, Lee W. Cohnstaedt, Corey L. Brelsfoard

**Affiliations:** 1 Department of Biological Sciences, Texas Tech University, 2901 Main St., Lubbock, TX 79409 USA; 2 USDA-ARS Arthropod Borne Animal Disease Research Unit, 1515 College Ave., Manhattan, KS 66502 USA

**Keywords:** *Culicoides sonorensis*, *Wolbachia pipientis*, Population replacement, Population suppression, Biting midge

## Abstract

**Background:**

Biting midges of the genus *Culicoides* vector multiple veterinary pathogens and are difficult to control. Endosymbionts particularly *Wolbachia pipientis* may offer an alternative to control populations of *Culicoides* and/or impact disease transmission in the form of population suppression or replacement strategies.

**Methods:**

*Culicoides sonorensis* cell lines were transfected with a *Wolbachia* infection using a modified shell vial technique. Infections were confirmed using PCR and cell localization using fluorescent *in situ* hybridization (FISH). The stability of *Wolbachia* infections and density was determined by qPCR. qPCR was also used to examine immune genes in the IMD, Toll and JACK/STAT pathways to determine if *Wolbachia* were associated with an immune response in infected cells.

**Results:**

Here we have transfected two *Culicoides sonorensis* cell lines (W3 and W8) with a *Wolbachia* infection (*w*albB) from donor *Aedes albopictus* Aa23 cells. PCR and FISH showed the presence of *Wolbachia* infections in both *C. sonorensis* cell lines. Infection densities were higher in the W8 cell lines when compared to W3. In stably infected cells, genes in the immune Toll, IMD and JAK/STAT pathways were upregulated, along with Attacin and an Attacin-like anti-microbial peptides.

**Conclusions:**

The successful introduction of *Wolbachia* infections in *C. sonorensis* cell lines and the upregulation of immune genes, suggest the utility of using *Wolbachia* for a population replacement and/or population suppression approach to limit the transmission of *C. sonorensis* vectored diseases. Results support the further investigation of *Wolbachia* induced pathogen inhibitory effects in *Wolbachia*-infected *C. sonorensis* cell lines and the introduction of *Wolbachia* into *C. sonorensis* adults *via* embryonic microinjection to examine for reproductive phenotypes and host fitness effects of a novel *Wolbachia* infection.

## Background

*Culicoides* species are small hematophagous insects that have been shown to harbor more than 50 different viruses of veterinary and medical importance [1]. These viruses include orbiviruses, such as African horse sickness virus (AHSV), Schmallenberg virus (SBV), bluetongue virus (BTV) and epizootic hemorrhagic disease virus (EHDV), which significantly impact deer and livestock production through loss of profits and trade restrictions [1, 2]. Multiple outbreaks of blue tongue virus (BTV) of different serotypes, topotypes (regional variants of particular serotypes) and strains have been recorded in Europe in recent decades [3, 4]. One of the largest European outbreaks to date recorded in the Netherlands, resulted in economic damage greater than $150 million dollars [5]. The circulation of established and newly established BTV serotypes still continues to affect large areas of southern and eastern Europe. Currently, there are at least 11 invasive BTV serotypes circulating in the USA [6–10] and the number of serotypes in the USA is on the rise, suggesting the epidemiology of BTV is changing and could result in extensive disease in USA livestock if the virus were to infect naive host populations [11]. Worldwide estimates of direct and indirect losses due to BTV have been estimated to top $3 billion dollars [12].

*Culicoides-*vectored arborvirus and zoonotic diseases have limited methods of treatment and prevention and rely on inadequate forms of vector control to combat the spread of the disease [2, 13, 14]. Current control methods for *Culicoides* are focused on treating livestock with topical insecticides at livestock production facilities and farms, but are typically met with limited success, depending on the *Culicoides* species targeted [2, 13, 14]. Furthermore, little is known about the biology of many *Culicoides* species, specifically immature habitat selection, making the effective application of insecticides to control immatures difficult [13, 15]. Habitat modification to remove standing water and removal of manure is often used to impact populations of *Culicoides* near livestock, but is limited to use in areas near livestock production. The combination of larvicide and adulticidal treatments have also demonstrated some success, but the true efficiency of this type of control has not been assessed, and this type of treatment typically does not reduce the numbers of *Culicoides* adults, if only treating around a farm property [16, 17]. Vaccines are available for a few *Culicoides-*transmitted viruses. Active virus vaccines are available for BTV serotypes, but are limited in effectiveness due to the large number of serotypes and the potential for genome segment re-assortment of the BTV [18]. Inactivated vaccines are currently available for -BTV and have been used in Europe. However, inactivated vaccines are expensive and not an effective solution when treating large amounts of livestock [19, 20]. Therefore, alternative control measures are needed to supplement the few existing control measures for *Culicoides* species.

*Wolbachia pipientis* may offer an alternative environmentally friendly control measure for *Culicoides* midges and the pathogens they vector. *Wolbachia* is an obligate intracellular bacterium found in > 55% of insects, as well as filarial nematodes and terrestrial crustaceans [21, 22]. In insects, *Wolbachia* causes alterations in host reproduction, with several phenotypes including feminization, parthenogenesis, male killing and cytoplasmic incompatibility (CI) [23]. Recently, *Wolbachia* has been used as a strategy for mosquito suppression and disease control and has become a topic of global relevance [24, 25]. Two *Wolbachia*-based strategies are currently being implemented in the field for mosquito and disease control. The first is a *Wolbachia* incompatible insect technique (IIT) approach based on mass inundative releases of incompatible male mosquitoes similar to the Sterile Insect Technique (SIT), with the goal of suppressing natural populations through sterile mattings [26, 27]. The second is based on the discovery that some *Wolbachia* interfere with viruses and other microbes in the same host [28–32]. Particular *Wolbachia* variants (e.g. the *w*Mel strain) can block dengue virus transmission without impacting *Aedes aegypti* fitness [28]. In addition, *Wolbachia* has also been shown to impact chikungunya virus, Zika virus and the yellow fever virus in their mosquito host [28–32]. Because *Wolbachia*-infected females can mate and produce viable offspring with infected and uninfected males alike, and infected males when mated with uninfected female produce non-viable offspring, the resulting reproductive advantage of *Wolbachia* infected individuals can drive a given disease refractory phenotype into a natural population limiting disease transmission.

Biological control using *Wolbachia* has been limited to disease vector mosquitoes, presumably because of the immediate need for novel tools for hard to control mosquito species such as *Ae. aegypti* and *Aedes albopictus*, that are primary and secondary vectors of dengue and Zika viruses, respectively. The success of the recent field applications of *Wolbachia* control techniques in mosquitoes suggests the potential for the transition of this technology to other insect disease vectors of human and zoological importance. *Wolbachia* infections have recently been confirmed in multiple populations and species of *Culicoides* midges in Europe and Australia [33, 34], suggesting that introducing novel *Wolbachia* infection types into uninfected *Culicoides* species is possible. Furthermore, the existence of natural infections suggests that reproductive phenotypes such as cytoplasmic incompatibility could exist in natural populations that harbor *Wolbachia* infections. Here, we demonstrate that *Wolbachia* infections can be introduced into *Culicoides sonorensis* cell lines as an initial step towards the investigation of a *Wolbachia* based control strategy for *Culicoides* midges. Recent studies have suggested that *Wolbachia* infections from a donor host can be pre-adapted to a target host cell lines in order to facilitate the adaptation for survival in a novel host [35]. The successful transfection of *Wolbachia* in *Culicoides sonorensis* cell culture may be a precursor to the successful germ line transfection of *C. sonorensis*. Furthermore, we use transfected cell lines to investigate a host immune response associated with a *Wolbachia* infection, which could suggest a reduced ability of *Wolbachia* infected *C. sonorensis* to transmit viral pathogens.

## Methods

### Cell culture

Aa23 *Ae. albopictus* cell lines were cultured in 75 cm^2^ cell culture flasks (TPP™, Techno Plastic Products, Trasadingen, Switzerland) in Schneider’s insect medium (SM) (MilliporeSigma, St. Louis, MO, USA) supplemented with 10% fetal bovine serum (FBS) (Table 1) [36]. W8 and W3 *C. sonorensis* cells were cultured in 25 cm^2^ cell culture flasks (TPP™, Techno Plastic Products) in SM (24.5 g/l) supplemented with 0.4 g/l sodium bicarbonate, 0.0585 g/l l-glutatmine, 0.006 g/l reduced glutathione, 0.03 g/l l-Asparagine, 18 µl of 10 mg/l Bovine insulin and 5% FBS (Table 1). All cell cultures were incubated at 28 °C with a CO_2_ concentration of 0.2%.

**Table 1:** Mosquito and midge cell lines used in the transfection experiments

Cell line	Host	Natural infection status	Transfected *Wolbachia* type	Reference
Aa23	*Aedes albopictus*	*wa*lbB	N/A	Dobson et al. [36]
W8—(CuVa-W8a)	*Culicoides sonorensis*	Uninfected	*wa*lbB	McHolland & Mecham [38]
W3—(CuVa-W3)	*Culicoides sonorensis*	Uninfected	*wa*lbB	McHolland & Mecham [38]

### *Wolbachia* isolation and transfection experiments

The *walbB* infection was isolated from the Aa23 cells grown to > 95% confluency. Extracellular *Wolbachia* was isolated using a modified procedure as previously described [37]. The adherent cells were scraped, and the cell culture was centrifuged at 2500× *g* at 4 °C for 10 min. The supernatant was removed, and the pellet was re-suspended in 6–8 ml of a SM + 10% FBS solution. Next, 3 mm glass beads were added to the solution up to the three ml mark in a 15-ml centrifuge tube and vortexed at high speed for 5 min, followed by centrifugation at 2500× *g* at 4 °C for 10 min. The supernatant was then passed through a 5-μm syringe filter and the resulting solution was split into 1.5 ml centrifuge tubes (approximately 1 ml per tube) and subjected to sucrose gradient centrifugation (200 mM sucrose solution) at 17,000× *g* for 10 min at 4 °C. The resulting pellet was re-suspended in SM with 10% FBS and passed through a 2.7-μm syringe filter to collect extracellular *Wolbachia*. To examine for contamination of extracts with Aa23 cells, environmental bacteria, and/or fungi, a portion of the extract was added to Schneider’s media with 10% FBS in a 25-cm^2^ flask and incubated at 28 °C with a CO_2_ concentration of 0.2%.

**Fig. 1:**
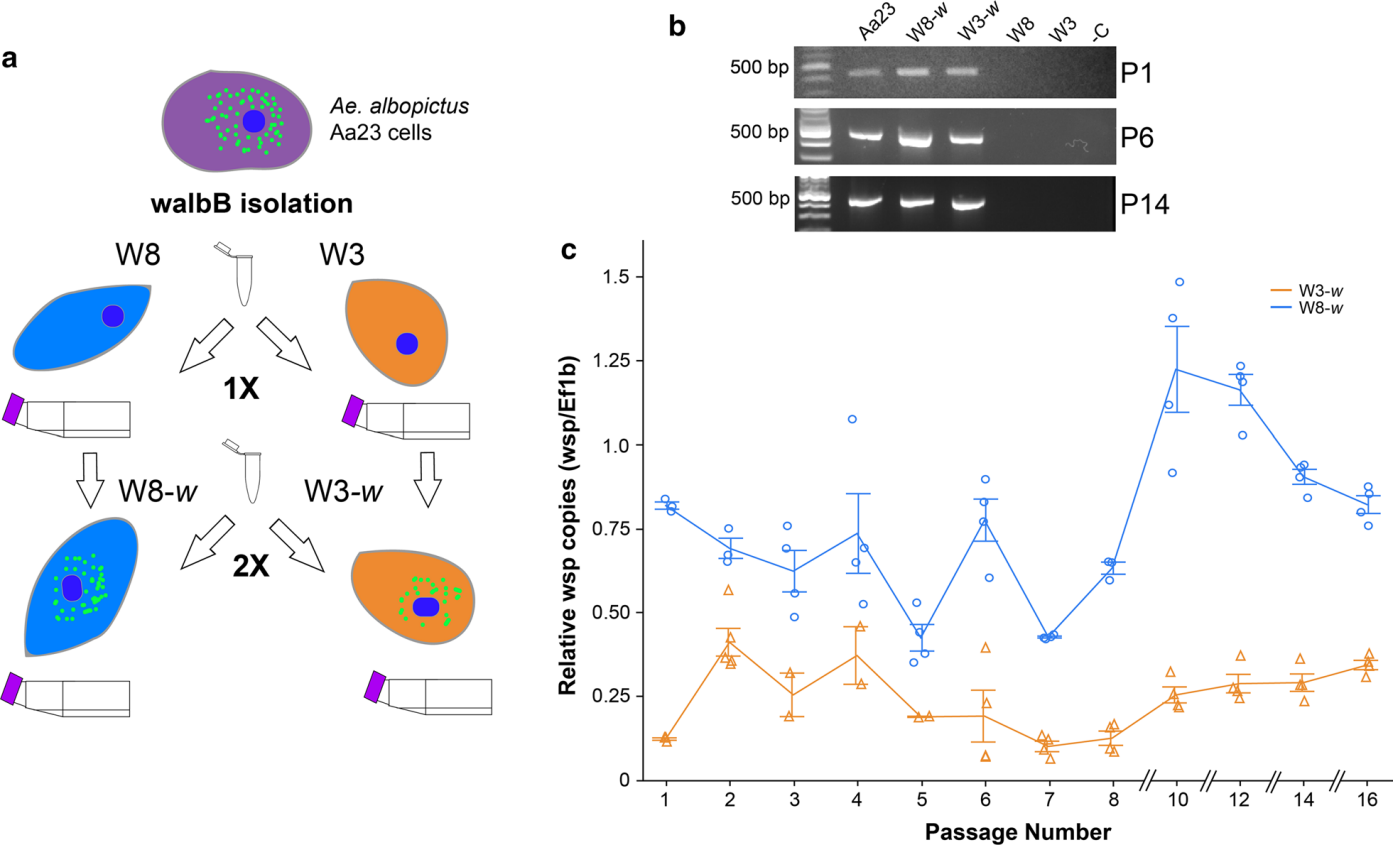
**a** *Wolbachia* transfection procedure using *Wolbachia* isolated from *Ae. albopictus* Aa23 donor cells to transfect W8 and W3 *C. sonorensis* cell lines. 1× and 2× refer to the two rounds of the transfection procedure to generate *Wolbachia* infected W8-*w* and W3-*w* cell lines. **b** PCR confirmation of *Wolbachia* infections in Aa23, W8-*w*, W3-*w* transfected cell lines and absence of infections in W8 and W3 cell lines at passages 1, 6, and 14. **c** *Wolbachia* density as determined by qPCR of W3-*w* and W8-*w* cell lines for 16 passages. Data are represented as the mean ± standard error (SEM)

Infection of W8 and W3 cell lines was carried out using a modified shell vial technique [36]. Briefly, shell vials (29 × 80 mm) were seeded with W8 or W3 cells at 90% confluency and allowed to adhere to vial surface for two hours. Isolated extracellular *Wolbachi*a was applied to W8 or W3 cells. The shell vial was centrifuged at 2500× *g* for 40 min at 20 °C and the cells transferred to a 25-cm^2^ flask with 5 ml of fresh media. In an attempt to increase *Wolbachia* infection rates, a second shell vial experiment was performed on the previously transfected W8-*w* and W3-*w* cells (2×) (Fig. 1a). One day after infection, the cells were transferred into a 25-cm^2^ flask containing 5 ml of fresh medium. W3-*w* and W8-*w* cells were passaged every 6–8 days at a ratio of 1:4 (cell culture: new media).

### PCR testing of *Wolbachia* infections

To confirm the *Wolbachia* infection was successfully isolated from the Aa23 cells in passage 1 and to confirm establishment in W3-*w* and W8-*w* cell lines, DNA was isolated from passage 1, 6 and 14 of 2× *Wolbachia* infected W3-*w*, W8-*w* and uninfected W3 and W8 and Aa23 cells using a Qiagen DNeasy Blood and Tissue Kit (Qiagen, Hilden, Germany). For detection of *Wolbachia,* a PCR assay that amplified a 438 bp *16S* rRNA gene fragment was used with the specific primer set wspecF and wspecR [38] (Additional file 1: Table S1). For all RXNs, 1 µl of isolated DNA was amplified in 25 mM KCL, 25 mM Tris–HCL (pH 9.0), 20 mM (NH_4_)_2_SO_4_, and 0.025% Triton X-100, 0.25 mM MgCl_2_, 0.25 mM dNTPs, 0.5 mM primers, and 1 U of Taq DNA polymerase in a total volume of 25 µl. The PCR amplification protocol was 10 min at 95 °C, 35 cycles of 30 s at 95 °C, 30 s at 54 °C and 1 min at 72 °C, followed by a 10 min extension step at 72 °C using a T100 Thermocycler (Bio-Rad, Hercules, CA, USA). A volume of 5–10 µl of each amplicon was separated on 1.5% agarose gel, stained with GelRed (Biotium, Hayward, CA, USA) and visualized under ultraviolet illumination.

### Fluorescence *in situ* hybridization

Fluorescence *in situ* hybridization (FISH) was performed on the W8-*w* and W3-*w* and W8 and W3 cell lines to confirm the presence and absence of *Wolbachia,* respectively. For the FISH procedures, W8-*w* and W3-*w* cells were at passage 4 post-*Wolbachia* infection. Cells were grown to 90% confluency at 28 °C and 300 µl of the cells were added to an 8-well Nunc® Lab-Tek^®^ Chamber slide system (Thermo Fisher Scientific, Waltham, MA, USA). The cells were incubated in the chambered wells overnight for ~ 15 h at 28 °C. Cells were fixed in 4% formaldehyde (in 1× PBS) for 40 min at room temperature (RT) and followed by two washes with 1× PBS-T. Next, the cells were pre-hybridized for ~ 2 h at RT. The pre-hybridization buffer consisted of 50% deionized formamide, 20% 20× sodium saline citrate (SSC) solution, 1% 50× Denhardt’s Reagent, 10% 1 mol dithriothreitol (DTT), 0.25 mg/ml t-RNA and 0.25 mg/ml poly(A). The pre-hybridization step was followed by an overnight hybridization (~ 18 h) at 37 °C in a moist environment with gentle shaking. The hybridization buffer consisted of pre-hybridization buffer supplemented with 200 mg/ml dextran sulfate, 250 mg/l salmon sperm DNA and *Wolbachia* specific probes (5′-/56-FAM/ AAT CCG GCC GAR CCG ACC C-3′); 5′-/56-FAM/ CTT CTG TGA GTA CCG TCA TTA TC-3′) [37]. After hybridization, the cells were washed with denatured SSC solution in the following order: wash buffer 1 (1× SSC augmented with 10 mmol/l DTT) at room temperature with gentle shaking, wash buffer 1 at 55 °C with gentle shaking, and two washes at 55 °C with wash buffer 2 (0.5× SSC augmented with 10 mmol/l DTT) with gentle shaking. Following the wash steps, cells were stained with DAPI at room temperature for 5 min followed by three 5-min washes with 1× PBS. The cells were then observed using a Nikon (Melville, NY, USA) A1 HD25/A1R HD25 confocal microscope with high-definition resonant scanner at a magnification of 20× and 60×. All images were processed using ImageJ and Adobe Photoshop (Adobe Systems, San Jose, CA, USA).

### *Wolbachia* quantification

qPCR was used to quantify the density of *Wolbachia* in W8-*w* and W3-*w* infected cell lines. DNA was extracted from *Wolbachia* infected cells using Qiagen DNeasy Kit. *Wolbachia* density was determined by amplifying a fragment of the *Wolbachia wsp* gene (Additional file 1: Table S1) [39–42] using Platinum SYBR Green qPCR SuperMix-UDG (Thermo Fisher Scientific) on a Applied Biosystems 7300 real time PCR system (Applied Biosystems, Beverly, MA, USA) and completed in duplicate or triplicate. The relative abundance of *Wolbachia* in W8-*w* and W3-*w* cell lines were normalized to the single copy elongation factor 1b gene (Additional file 1: Table S1).

### Immune response experiments

Qiagen RNeasy Mini Kit was used to isolate RNA for quantification of host cell gene expression (Qiagen). Isolated RNA from W8-*w* cells was treated with DNase and cDNA synthesized using an NEVB LunaScript RT superMix kit (New England Biolabs, Ipswich, MA, USA) following the manufacturerʼs guidelines. To determine the response of the immune pathways to the *w*albB infection in W8-*w* cell lines, qPCR was used to determine host gene expression of immune genes involved in the IMD, Toll and Jak/Stat pathways and anti-microbial peptides previously identified in *C. sonorensis* (Additional file 1: Table S1). All reactions were performed by amplifying the target immune genes using Platinum SYBR Green qPCR SuperMix-UDG, completed in triplicate and normalized to the elongation factor 1b gene using the 2^–∆∆ct^ method.

### Statistics

JMP software (SAS, Cary, NC, USA) was used for statistical analysis. Statistical significance of immune gene expression levels of *Wolbachia* infected and uninfected *Culicoides* cells was determined by t-tests, with a significance level of *P* < 0.05.

## Results

### Establishment and *Wolbachia* density in *Culicoides* cells

Prior to *Wolbachia* transfection procedures, W8 and W3 cell lines were confirmed for absence of a natural *Wolbachia* infection (Fig. 1a, b). Successful *Wolbachia* isolation from Aa23 cells was confirmed by a positive PCR in passage one post transfection (Fig. 1b). After two *Wolbachia* transfection procedures, W8-*w* and W3-*w* cell lines tested positive for *Wolbachia* infections using PCR at passages 4 and 16 (Fig. 1b). No contamination was observed in W8-*w* and W3-*w* cell lines post-transfection or in control flasks containing *Wolbachia* extract and cell culture media.

To investigate the infection dynamics of W8-*w* and W3-*w* cell lines, qPCR was used to determine *Wolbachia* density using isolated DNA samples from eight passages of W3-*w* and W8-*w* cell lines. W8-*w* consistently maintained a higher density *Wolbachia* infection than W3-*w* (Fig. 1c), but there was no evidence for *Wolbachia* infection loss in either cell lines, and infections appeared to be maintained at a relative density of approximately 0.77 ± 0.26 (mean ± standard deviation, SD) and 0.24 ± 0.12 (mean ± SD) for the W8-*w* and W3-*w* cell lines, respectively (Fig. 1c). After transfection with *w*albB, W8-*w* and W3-*w* cells were able to be cryogenically frozen in SM with 10% FBS and 10% DMSO. Cells were able to be re-established and harbor similar densities of *Wolbachia* infections compared to unfrozen original cell lines for both W3 and W8 cell lines.

### Localization of walbB in *Culicoides* cells by FISH

**Fig. 2:**
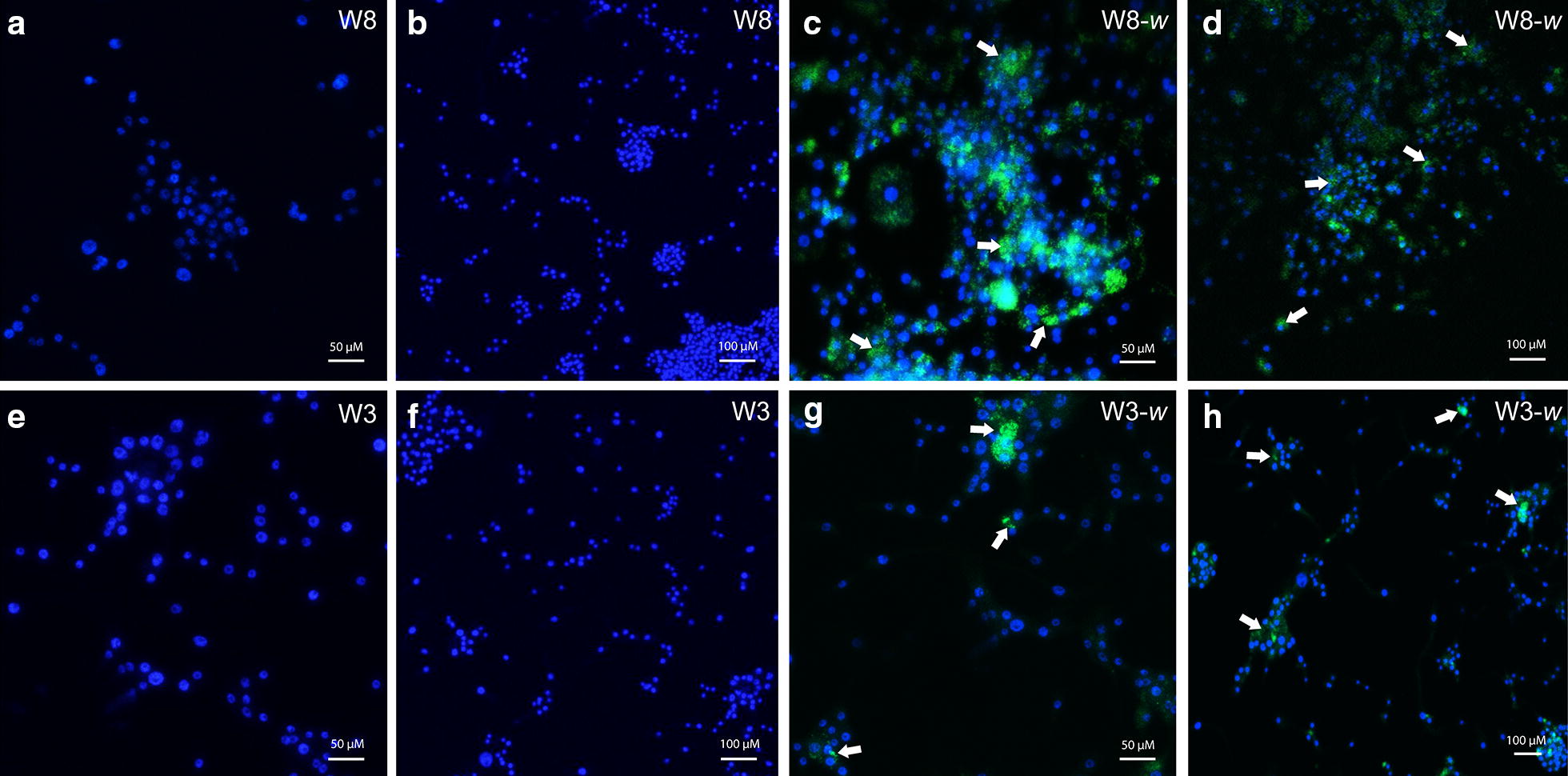
Fluorescent *in situ* hybridization of uninfected (**a**, **b**) and *Wolbachia-*infected W8-*w* cells (**c**, **d**) at 60× and 20× magnifications, respectively. FISH of uninfected (**e**, **f**) and *Wolbachia*-infected W3-*w* cells (**g**, **h**) at 60× and 20× magnifications, respectively. Cell nuclei are shown in blue and *Wolbachia* specific probes are shown in green. White arrows indicate the presence of *Wolbachia* in cells

*Wolbachia* specific fluorescently labeled oligonucleotides were used to target the *Wolbachia wsp* gene in the *Culicoides* cells. No fluorescent signal was observed in the uninfected *Culicoides sonorensis* (Fig. 2). Fluorescent microscopy observations showed the presence of *Wolbachia* in the cytoplasm of the W8-*w* and W3-*w* cell lines. However, the limited level of hybridization suggested a low level of infection in the W3-*w* cell lines, while FISH in the w8-*w* cell line suggested a higher density infection compared to W3-*w* (Fig. 2).

### *Wolbachia* effects on immune gene expression in *Wolbachia* infected *Culicoides* cells

**Fig. 3:**
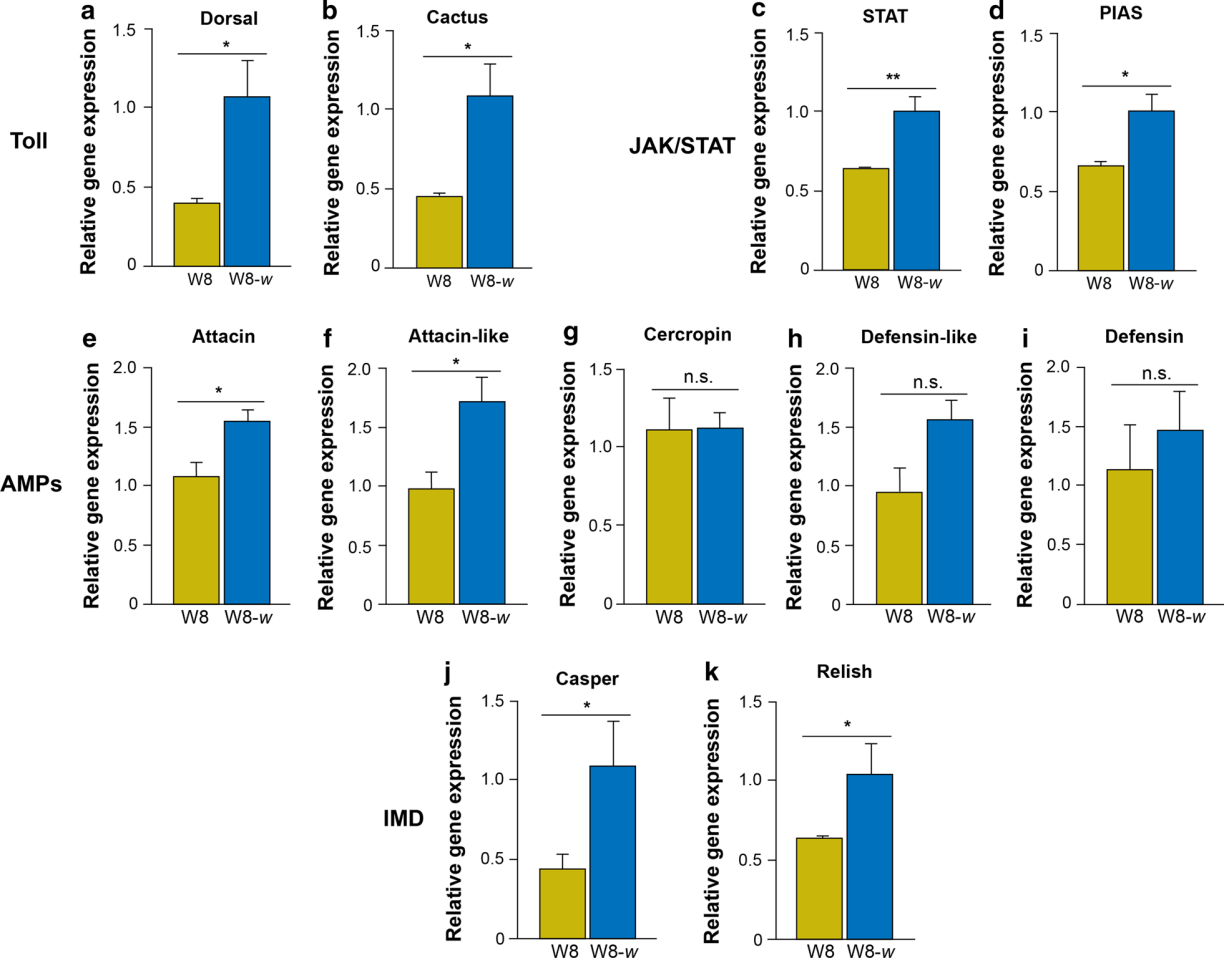
Immune response after establishment of the *w*albB *Wolbachia* infection in the W8-*w* cell line, Toll pathway regulators Dorsal (**a**) and Cactus (**b**), JAK/Stat pathway regulators STAT (**c**), PIAS (**d**), anti-microbial peptides (AMP) Attacin (**e**), Attacin-like (**f**), Cercropin (**g**), Defensin-like (**h**), Defensin (**i**) and IMD pathway regulators Caspar (**j**) and Relish (**k**). Data are represented as the mean ± standard error (SEM) of two or three biological replicates. *P < 0.05, **P < 0.01, n.s., not significant (*P* > 0.05)

After the establishment of a *Wolbachia* infection in the W3-*w* and W8-*w* cell lines at passage 5, we examined the gene expression of immune response in only the W8-*w* cell line due to the higher density of the *w*albB infection. To examine for an interaction of *w*albB and the host cell immune response, we performed qPCR on selected genes in the JAK-STAT, IMD and Toll pathways. In addition, we examined the expression of other immune-related genes including antimicrobial peptides (AMPs). W8-*w* infected cells had a significant increase in immune gene expression for STAT (t-test; *t* = 4.14, *df* = 3, *P* = 0.01), PIAS (t-test; *t* = 3.18, *df* = 3, *P* = 0.02), Caspar (t-test; *t* = 3.22, *df* = 3, *P* = 0.04), Relish (t-test; *t* = 2.02, *df* = 3, *P* = 0.05), Dorsal (t-test; *t* = 3.01, *df* = 3, P = 0.02), Cactus (t-test; *t* = 3.04, *df* = 3, *P* = 0.03), Attacin (t-test; *t* = 3.16, *df* = 3, *P* = 0.03) and Attacin-like genes (t-test; *t* = 2.81, *df* = 3, *P* = 0.05) when compared to W8 uninfected cells (Fig. 3). Cercropin (t-test; *t* = 0.05, *df* = 3, *P* = 0.48), Defensin (t-test; *t* = 0.66, *df* = 4, *P* = 0.27) and Defensin-like (t-test, *t* = 2.32, *df* = 4, *P* = 0.06) showed no significant difference in immune gene expression when compared to the uninfected W8 cells (Fig. 3).

## Discussion

The generation of cell lines with novel *Wolbachia* infections is a logical first step towards investigating whether an infection can be established in a novel insect host. Transfer of *Wolbachia* to novel hosts is time consuming and difficult using microinjection techniques to introduce *Wolbachia* infections into insect embryos, and more often than not, attempts are met with limited success. *Wolbachia* establishment in cell lines has also been shown as a way to pre-adapt a *Wolbachia* infection to a novel host potentially leading to a more successful transfection [35, 38]. The generation of two novel *Wolbachia-*infected *C. sonorensis* cell lines (W8-*w* and W3-*w*) is a notable advancement towards generating novel *Wolbachia* infections in adult midges.

The observed *Wolbachia* infection densities in the W8-*w* and W3-*w* cell lines are lower than previously reported for *Drosophila*, mosquito and sand fly host cells [35, 43, 44]. While higher *Wolbachia* infection densities have been reported to result in pathogen blocking and cytoplasmic incompatibility [41, 45, 46], other studies have suggested that low level infections can also result in cytoplasmic incompatibility (CI) and possibly pathogen blocking [47, 48]. Few studies have examined for *Wolbachia* infections in native *Culicoides* spp. populations, and those that have, demonstrated that *Culicoides* species have low density *Wolbachia* infections *in vivo* and a low prevalence in natural populations [33, 34]. Detection of *Wolbachia* infections in *Culicoides* spp. collected in Australia was accomplished by using qPCR over less sensitive conventional PCR [33]. *Wolbachia* infections have also been noted in *Culicoides* spp. collected in Spain, albeit at a low prevalence in populations, which could again be associated with a low *Wolbachia* density in adult host midges and a low detection level [34]. Hence, the low densities of *Wolbachia* observed in the W8-*w* and W3-*w* cell lines may be common to *Culicoides*. This observed result could be a function of the ability of *Wolbachia* to upregulate the immune system in its *Culicoides* host and inhibiting *Wolbachia* infection proliferation, or could be the result of environmental parameters such as temperature [49–51], host age [50, 52], sex [52] or *Wolbachia* strain [53], which have been demonstrated to impact *Wolbachia* density in its insect host. It is currently unclear why the W8-*w* cell line is able to maintain a higher *Wolbachia* density than the W3-*w* cell line, but this observation could be related to predominant cell morphology and type of each culture. The W3 cell line is predominantly, epithelial-like cells that are firmly adherent [38]. The W8 cell line contains predominantly fusiform or stellate shaped cells, which are lightly adherent [38]. Previous studies have shown *Wolbachia* can infect multiple cell and tissue types at different rates and densities [54].

The W8-*w* and W3-*w* cell lines were infected with a *w*albB infection from donor Aa23 *Ae. albopictus* cells. The *w*albB infection type was an optimal candidate for *C. sonorensis* cell line transfection because: (i) the *w*albB infection has been shown to fall within the same B-clade as other *Wolbachia* infections reported in multiple *Culicoides* spp. collected in native populations in Spain [34], suggesting a higher likelihood for a successful transfection; and (ii) the *w*AlbB infection has shown parasite and virus inhibitory effects in multiple insect vectors of disease. In *Anopheles stephensi*, *w*albB has been demonstrated to reduce *Plasmodium* parasite development [55, 56]. Also, *w*albB has been demonstrated to suppress filarial worm loads and provide resistance to dengue virus transmission in *Aedes polynesiensis* [57].

While parasite and pathogen inhibitory effects have been reported in multiple *Wolbachia* insect systems, the mechanism of pathogen inhibition in *Wolbachia* infected insect hosts is not well understood [58–60]. Currently, there are two hypotheses proposed to understand *Wolbachia* induced pathogen inhibition. The first is that *Wolbachia* primes the host immune system, so when the pathogen enters the insect host, invasion is inhibited. Second, *Wolbachia* is hypothesized to be competing for metabolic components such as amino acids and cholesterol with the host insect and the pathogen [61, 62]. For example, *Wolbachia* replication is known to be dependent upon host cell cholesterol production and requires cholesterol-rich host membranes to form the vacuole surrounding each bacterium, which is hypothesized to lead to competition for cholesterol between *Wolbachia* and pathogens [61, 63]. To test the first hypothesis, we examined the expression of immune related genes in the IMD, Toll and JAk/STAT pathways in the W8-*w* cell line. Unfortunately, knowledge of immune pathways in *C. sonorensis* is limited, but several immune genes have been identified and characterized in a previous study [64]. Furthermore, the availability of the *C. sonorensis* annotated genome (GenBank: GCA_900258525) allowed for the identification of genes in Toll, JAK/STAT and IMD pathways [65]. The upregulation of Dorsal, Cactus, STAT, PIAS, Caspar, RELISH, Attacin, and an Attacin-like anti-microbial peptides suggest that *Wolbachia* can affect the *C. sonorensis* immune system pathways in different cascades. Perhaps, this priming of the immune system could have an effect on orbivirus proliferation in its *Culicoides* host. Previous transcriptome studies have demonstrated 165 genes, including genes in the Toll and IMD pathways and AMPs that were differentially expressed between vector competent or refractory *C. sonorensis* when challenged with a BTV infection [65]. Future studies could include the inoculation of W8-*w* and W3-*w* cell lines with the orbiviruses BTV, AHSV, SBV, and/or EHDV and determining whether a novel *Wolbachia* infection can induce virus inhibitory effect in *C. sonorensis* cells. Both the W3 and W8 cell lines have previously been shown to be susceptible to BTV and EHDV infection [38]. If pathogen viral inhibition is observed, additional gene families other than traditional gene pathways could also be investigated for their functional role in potential pathogen blocking [66].

Here we have only examined the effect of one *Wolbachia* infection type on *C. sonorensis* cells. Future studies could also include the transfection of W8 and W3 cell lines with alternative *Wolbachia* infections such as *w*Mel, which has also been demonstrated to provide virus inhibitory phenotypes in mosquitoes [46, 67]. It is also important to note that while the data presented suggest an upregulation in the immune system and a potential pathogen blocking effect, there is the possibility that a *Wolbachi*a and *Culicoides* host association facilitates a pathogen infection in *Culicoides* spp. Previous studies have demonstrated an increase in the ability of *Culex tarsalis* to transmit West Nile virus when infected with *Wolbachi*a and an enhanced flavivirus infection rate in *Ae. aegypti* [68, 69].

The W8-*w* and W3-*w* cell lines will continue to be passaged and maintained, with the goal of adapting the *w*albB infection to *C. sonorensis*. Subsequently, the *w*albB *Culicoides* adapted infection can be extracted from the W8-*w* or W3-*w* cell line and used for future microinjection experiments with the goal of generating a *w*albB germ line infection in *C. sonorensis* adults. If a stable germ line maternally inherited *Wolbachia* infection can be attained in *C. sonorensis* adults, further work would be needed to ascertain whether novel *Wolbachia* infections have an effect on *C. sonorensis* fitness, any reproductive phenotypes such as CI associated with *Wolbachia* infections, or any orbiviruses inhibitory effects. Further investigation is also needed to develop microinjection protocols for introducing *Wolbachia* infections into *C. sonorensis* embryos. This future work would open up exciting possibilities to investigate whether *Wolbachia*-based approaches could be used as an additional tool to control *C. sonorensis* and other *Culicoides* species and as an additional tool to limit transmission of veterinary important orbiviruses.

## Conclusions

Here, we were able to establish a *Wolbachia* infection in *C. sonorensis* cells (W8 and W3 cell lines) and that the W8 *Wolbachia* infected cell line demonstrated an upregulation of the Toll, IMD, JACK/STAT pathways and the production of anti-microbial peptides. The results suggest the potential utility of *Wolbachia*-based approaches for vector control strategies and to limit disease transmission by *C. sonorensis* and other *Culicoides* species of veterinary importance. Further investigation is needed to introduce germline infections into *C. sonorensis* and examine for *Wolbachia* induced reproductive phenotypes and pathogen inhibitory effects.

## Supplementary information

**Additional file 1: Table S1.** Primer sequences used to confirm *Wolbachia* infection status, determine *Wolbachia* density, and host immune pathway gene expression.

## Data Availability

The *Culicoides* cell cultures that support the findings of this study are available from the United States Department of Agriculture Agricultural Research Service Arthropod-borne Animal Diseases Research Unit, but restrictions apply to the availability of these cultures, which were used under license for the current study, and so are not publicly available. Data are however available from the authors upon reasonable request and with permission of Department of Agriculture Agricultural Research Service Arthropod-borne Animal Diseases Research Unit.

## Abbreviations

AHSV: African horse sickness virus
SBV: Schmallenberg virus
BTV: bluetongue virus
EHDV: epizootic hemorrhagic disease virus
CI: cytoplasmic incompatibility
IIT: incompatible insect technique
AMP: anti-microbial peptides
SM: Schneider’s media
FBS: fetal bovine serum
FISH: fluorescent *in situ* hybridization
PCR: polymerase chain reaction
qPCR: quantitative polymerase chain reaction
DTT: dithriothreitol
SCC: sodium saline citrate
RT: room temperature
PBS: phosphate-buffered saline
PBS-T: phosphate-buffered saline-Tween
