# Supplementary material for: Transfection of Culicoides sonorensis biting midge cell lines with Wolbachia pipientis
Source: Parasit Vectors. 2019 Oct 15;12:483. doi: 10.1186/s13071-019-3716-0 (PMC6792224; doi:10.1186/s13071-019-3716-0)
Supplement: Supplementary file 1 — Additional file 1: Table S1. Primer sequences used to confirm Wolbachia infection status, determine Wolbachia density, and host immune pathway gene expression. [file 13071_2019_3716_MOESM1_ESM.pdf]

Additional file 1: Table S1. Primer sequences used to confirm *Wolbachia* infection status, determine *Wolbachia* density, and host immune pathway gene expression.

Name	Primer sequence	Gene	Accession number	Reference
wspecF	CATACCTATTCGAAGGGATAG	16s rRNA	-	Werren and Windsor 2000
wspecR	AGCTTCGAGTGAAACCAATTC			
Wsp2-F	CATTGGTGTTGGTGTTGGTG	wsp	-	Sheehan et al. 2016
Wsp2-R	ACCGAAATAACGAGCTCCAG	wsp		
Relish-F	TTGAATGCCCCACTGAGTCC	Relish	GAWM01014884	This paper
Relish-R	TCTGAGCTTGCACCAACACT			
Caspar-F	GCATGTCACAAGTCAGCTCG	Caspar	GAWM01012793	This paper
Caspar-R	TCACTTCCGAGCAGTTGTCC			
Cactus-F	ACAAAGCCGTTGAGGATGGT	Cactus	GAWM01009580	This paper
Cactus-R	AGTCCTGCATATGTGGTGGC			
Dorsal-F	ATCCCACCATTCAAGTGCGT	Dorsal	GAWM01010294	This paper
Dorsal-R	TTGTGAGGATGCGGTCTGTG			
STAT-F	TGCATGGGTCAATGAGGGTG	STAT	GAWM01013279	This paper
STAT-R	ACGATCTGAGAGACTGCGTG			
PIAS-F	TCCAGACTCGGGCACTAGAA	PIAS	GAWM01011450	This paper
PIAS-R	TGCCGATACAGTTGCTCCAG			
Attacin-like-F	CATGGATTCAAGACCTCATCG	Attacin-like	GAWM0100844	Nayduch et al. 2014
Attacin-like-R	CCAAGACCTACTCCAGTTGTA			
Attacin-F	GGATTGTCGGGTAGTGTAAGT	Attacin	GAWM01017969	Nayduch et al. 2014
Attacin-R	CCAAAGTGCTGTGTTGATCTC			
Cecropin-F	AGCTCCTAGATGGAAAGGATG	Cercropin	GAWM01000005	Nayduch et al. 2014
Cecropin-R	CTTTGTATCCTGCAACGACTG			
Defensin_1-F	CAGACCAAATCCAAATCTCTCC	Defensin	GAWM01019039	Nayduch et al. 2014
Defensin_1-R	GGCAGTATCCTGACTTGTACT			
Defensin_2-F	TTCAACCAAGACTTTCATGCC	Defensin	GAWM01019040	Nayduch et al. 2014
Defensin_2-R	CAAGTTCCATTACGACAATAGCC			
EF 1b-F	ATCCGTGAAGAACGTCTCAAA	Elongation factor 1b	GAWM01010754	Nayduch et al. 2014
EF 1b-R	CATGGCTTAACTTCGAGGATG			
